# *Wolbachia* strain *w*AlbB confers both fitness costs and benefit on *Anopheles stephensi*

**DOI:** 10.1186/1756-3305-7-336

**Published:** 2014-07-21

**Authors:** Deepak Joshi, Michael J McFadden, David Bevins, Fengrui Zhang, Zhiyong Xi

**Affiliations:** 1 Department of Microbiology and Molecular Genetics, Michigan State University, East Lansing, MI 48824, USA; 2 Sun Yat-sen University - Michigan State University Joint Center of Vector Control for Tropical Diseases, Guangzhou, Guangdong 510080, China

**Keywords:** *Wolbachia*, Malaria, *Anopheles*, Fitness

## Abstract

**Background:**

*Wolbachia* is a maternally transmitted intracellular bacterium that is estimated to infect up to 65% of insect species, but it is not naturally present in *Anopheles* malaria vectors. *Wolbachia*-based strategies for malaria vector control can be developed either through population replacement to reduce vectorial capacity or through population suppression to reduce the mosquito population. We have previously generated *An. stephensi* mosquitoes carrying a stable *w*AlbB *Wolbachia* infection and have demonstrated their ability to invade wild-type laboratory populations and confer resistance to *Plasmodium* on these populations.

**Methods:**

We assessed *w*AlbB-associated fitness by comparing the female fecundity, immature development and survivorship, body size, male mating competiveness, and adult longevity of the infected *An. stephensi* to that of wild-type mosquitoes.

**Results:**

We found that *w*AlbB reduced female fecundity and caused a minor decrease in male mating competiveness. We also observed that wAlbB increased the life span of both male and female mosquitoes when they were maintained solely on sugar meals; however, there was no impact on the life span of blood-fed females. In addition, *w*AlbB did not influence either immature development and survivorship or adult body sizes.

**Conclusions:**

These results provide significant support for developing *Wolbachia*-based strategies for malaria vector control.

## Background

*Wolbachia* is a maternally transmitted intracellular bacterium that infects an estimated 65% of insect species [1,2] and 28% of mosquito species [3]. With such a broad host range, however, *Wolbachia* does not naturally infect the *Anopheles* malaria vectors or the primary dengue vector, *Aedes aegypti*. As a symbiotic bacterium of insects, *Wolbachia* can manipulate host reproduction in a number of “selfish” ways [2], resulting in its own spread into a population. Among these manipulations, cytoplasmic incompatibility (CI) is the most common and is the only phenotype observed in mosquitoes [4]. CI results in early embryonic death when an uninfected female mates with an infected male. An infected female can produce infected, viable offspring when she mates with either an infected or an uninfected male, but an uninfected female can produce viable offspring only when she mates with an uninfected male. Therefore, CI provides a reproductive advantage to *Wolbachia*-infected females over uninfected females, resulting in spread of *Wolbachia* into populations [5-7].

As a reproductive parasite, *Wolbachia* is typically highly enriched in insect reproductive tissues such as ovaries and testes. Depending on the host and the length of the association, *Wolbachia* can also have a broad tissue distribution and be found in somatic tissues such as the midgut, fat body, and salivary gland [8]. This broad distribution has frequently been observed in a system involving recent *Wolbachia*/host association, and it has resulted in an alteration in the host’s local physiological environment such that it has become resistant to pathogen infection [7,9,10]. Since its discovery, this *Wolbachia*-mediated pathogen interference has been observed in both naturally and artificially infected insect hosts and has been demonstrated in a broad spectrum of microorganisms, including dengue virus, Chikungunya, *Plasmodium,* and filarial worms [9,11-13].

The ability of *Wolbachia* to induce pathogen interference and also spread into mosquito vector populations makes it a potential biological agent for controlling both malaria and dengue [5,6,9,10,14]. The success of a recent field trial has indicated that *Wolbachia* can be deployed as a practical intervention strategy to control mosquito-borne diseases, with the potential for area-wide implementation [6]. With the stable introduction of *w*AlbB *Wolbachia* into *An. stephensi*[7], we are now able to develop a strategy of this kind for malaria vector control. Toward this end, we have previously verified that *w*AlbB can confer some resistance to the human malaria parasite *Plasmodium falciparum* in the transinfected *An. stephensi* LB1 strain [7]. Seeding of naturally uninfected *An. stephensi* populations with infected females results in *Wolbachia* invasion of laboratory mosquito populations [7].

A mathematical model has predicted that the strength of the CI, maternal transmission efficacy, and *Wolbachia*-associated fitness are three key parameters that determine the dynamics of *Wolbachia* in the population replacement process [15]. *w*AlbB displays perfect maternal transmission and induces nearly complete CI in *An. stephensi*, but it also produces a reduced egg hatch rate in LB1 mosquitoes [7]. This reduction in egg hatch was not observed in the other two mosquito species carrying *w*AlbB, the native host *Ae. albopictus* and the transinfected *Ae. aegypti* line [5,16]. In fact, *Wolbachia* was observed to produce a fitness benefit in these two species. In *Ae. albopictus*, *Wolbachia* (*w*AlbA and *w*AlbB)-infected females live longer, produce more eggs, and have higher hatch rates than do uninfected females [16]. An increase in life span was also observed in the transinfected *Ae. aegypti* females carrying *w*AlbB [10], and the transinfected *Ae. albopictus* males carrying *w*Mel [17]; however, *w*MelPop reduced longevity in both native *Drosophila* species and transinfected *Aedes* mosquitoes [18,19].

The ability of *w*AlbB to induce nearly complete CI when an LB1 male mates with a wild-type female supports the feasibility of using *Wolbachia* in a population suppression strategy. Derived from sterile insect technique (SIT), this strategy is also referred to as the incompatible insect technique (IIT) strategy, in which mass release of *Wolbachia*-infected males is used to induce CI matings with the wild-type females [20]. Success with this strategy has resulted in the eradication of *Culex pipiens* fatigans in a village in Burma and a recent effort to control the Polynesian tiger mosquito *Ae. polynesiensis* to eliminate lymphatic filariasis in the South Pacific [21,22]. In this strategy, the mating performance of LB1 males relative to the wild-type males is one of the key factors that facilitate the local suppression or eradication of *An. stephensi*.

Developing both population replacement and population suppression strategies to control *An. stephensi* requires a better understanding of *Wolbachia*-associated fitness in this mosquito species. In the present study, we compared the female fecundity, immature development and survivorship, body size, male mating competiveness, and adult longevity of infected LB1 and wild-type LIS mosquitoes. The results showed that *w*AlbB *Wolbachia* induces a fitness cost and also confers benefit on *An. stephensi*, underscoring the complexity of *Wolbachia*-*Anopheles* mosquito interactions.

## Methods

### Ethics statement

This study was carried out in strict accordance with the recommendations in the Guide for the Care and Use of Laboratory Animals of the National Institutes of Health. The protocols (03/14-036-00) were approved by the Michigan State University Institutional Animal Care and Use Committee.

### Mosquito lines

The wild-type *An. stephensi* (Liston strain [LIS]) mosquitoes were provided by the Johns Hopkins Malaria Research Institute. The *Wolbachia*-infected *An. stephensi* LB1 strain used in these experiments was artificially generated via embryonic injection and back-crossed with uninfected wild-type males for at least four generations to reduce genetic bottlenecks [7]. The aposymbiotic line LBT was derived from LB1 as described previously [7]. The adult mosquitoes were maintained on sugar solution at 27°C and 85% humidity with a 12-hr light/dark cycle according to standard rearing procedures. To initiate egg development, 5- to 7-day old adult females were fed on anesthetized BALB/c mice. Two days after blood-feeding, oviposition sites (cups containing filter paper moistened with water) were placed inside cages to harvest the eggs. After two consecutive nights of egg collection, eggs were hatched, and larval trays were set up. The larval rearing conditions used in the assessment of the life history traits were the same ones used for the stock lines, with the density at 100 larvae/660 ml water in all larval rearing pans. All the treatments on mosquitoes in the experiments described below were run from three trays of pupae independently. In the mosquito colonies, approximately 1,200 adults with the sex ratio 1:1 (female : male) were maintained in a cage (30 × 30 × 30 inches).

### Fecundity tests

Females were randomly selected from the population cages containing 8-to 9-day-old adult mosquitoes and then transferred to new cages. The mosquitoes in these cages were fed on anesthetized BALB/c mice for ~20 min or on commercial human or sheep blood, using a membrane feeding apparatus, for ~30 min. The unfed mosquitoes were then removed. Two days after blood-feeding, individual blood-fed females were transferred to a 50-ml Falcon tube with a bottom lining of moist filter paper supported by water-soaked cotton. After two nights of egg collection, egg papers were immersed in hatching cups (50-ml plastic cups half filled with water). The edges of the hatching cups were kept moistened with an extra lining of filter paper in order to prevent egg death, which is most likely to happen when eggs come into contact with dry cup edges. The eggs were left there for hatching for 2 days. The following day, the egg hatch was scored under a dissecting microscope.

### Assessment of life history traits

Approximately 100 larvae, hatched within 2 hr, were transferred to a plastic larval tray containing 660 ml of distilled water. The larval diet was composed of powdered fish food (First Bites, Hikari Tropical) and cat food (Purina cat food chow, Nestlé Purina PetCare). The mixture of the two foods was used only for 2 days, after which the cat food was used for the rest of the rearing period. The same amount of the larval diet was provided to both the infected and uninfected groups. Three biological replicates were performed for each group. Pupal development time in the trays was monitored and recorded. Pupae were collected at 8-hr intervals and transferred to 13-mm culture tubes. Later, emerging adults were sexed into males and females, and their emergence time was recorded.

### Wing size measurement

Wing sizes were used to estimate adult body sizes as described previously with a slight modification [23]. Adult mosquitoes that were nearly 7- 8 days old, post-eclosion, were killed with ethyl acetate. The wings were carefully broken with forceps at the apex of the alular region, dipped into the alcohol to remove folding, and mounted on a slide. The slides were then photographed under a microscope at 4× magnification. Linear measurement, from the humeral cross vein to the wing tip, excluding fringe, was conducted using Axio vision software (Carl Zeiss). Intact wings of adults from both the infected and uninfected groups were measured for size variation analysis.

### Mating competitiveness assays

Four adult cages were prepared with equal numbers of uninfected males and females (as outlined in Additional file 1: Table S1). Into these cages, varying numbers of infected males (0, 35, 100, or 200) were released so that the ratio of uninfected females: uninfected males: infected males was either 1:1:0, 1:1:1, 1:1:2 or 1:1:4. Mosquitoes were allowed to mate for 2 days. The mosquitoes were then blood-fed for approximately 20 min. Two days after blood-feeding, eggs cups were inserted into the cages for harvesting eggs. Eggs were collected for two nights. Egg hatching was then determined as described above. A second blood meal was given to the mosquitoes one week after the first feeding, and new collections of eggs were then made. The data from the two egg collections were pooled, and the egg hatch was measured and compared to an expected hatch rate, assuming equal competition between LB1 and LIS males [24].

### Life span assays

In the first experiment, the life span was measured when mosquitoes were maintained on sucrose alone. Twenty-five mosquitoes (1-2 days old), either males or females, were transferred to small cages designed from plastic bowls in which a 10% sucrose solution was available. In the second experiment, the female life span was measured after the mosquitoes were fed a blood meal. Females (4-5 days old) were fed on commercial human blood through a membrane feeding apparatus. The next day, the unfed females were removed, and ~ 40 blood-fed females were transferred to 20×20×20-cm cages. The mosquitoes were again allowed to take a blood meal; feeding was repeated at least four additional times, with a 6- to 7-day interval between each blood meal. For both experiments, dead mosquitoes were removed every day and recorded until no viable mosquitoes were left. Data from three replicate experiments were used for the survival assay.

### Statistical analysis

The results of experiments other than the mating competitive and survival assays were first checked for normality of distribution using the D’agostino and Pearson omnibus normality test. The results of this test were used to decide whether to use a parametric or a non-parametric test for further analysis. To compare the mating performances of the infected and uninfected males, chi-squared goodness-of-fit was used to analyze expected and observed hatchings in cages with varying ratios of males. Kaplan-Meier survival analysis (log-rank tests) was used for adult survivorship. GraphPad Prism version 5.00 for Windows was used for data analyses.

## Results

### *w*AlbB reduces fecundity in LB1 mosquitoes

**Figure 1:**
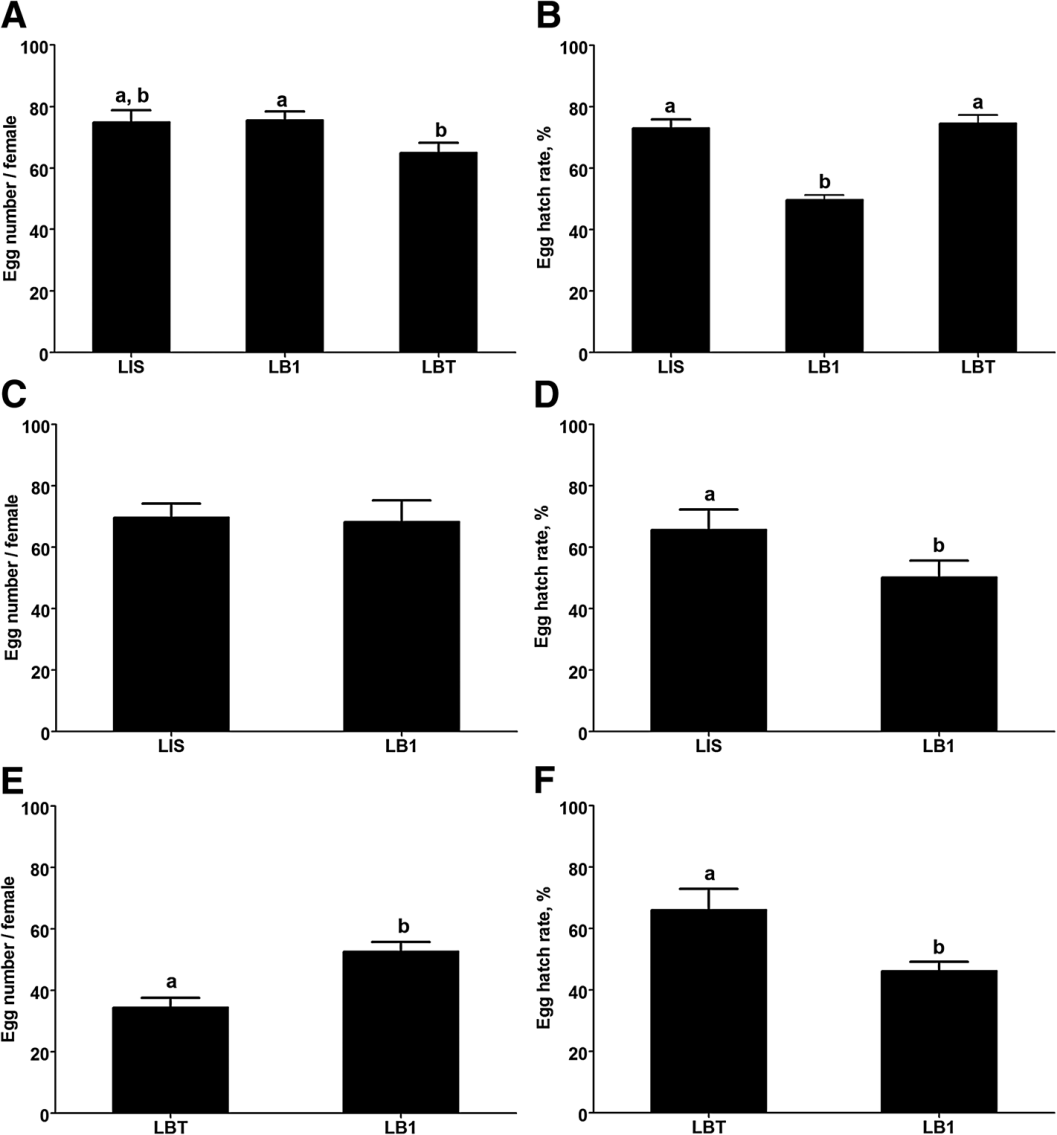
**Impact of *w*AlbB on *An. stephensi *fecundity.** The number of eggs laid by each individual female and the hatch rate after feeding on mouse **(A, B)**, sheep **(C, D)** or human **(E, F)** blood. For all figures, error bars represent standard error; statistical significance is represented by letters above each column, with different letters signifying distinct statistical groups [P < 0.05; Student’s *t*-test for **(A and E)**; P < 0.0001; chi-squared test for **(B, D and F)**].

In order to examine the impact of *w*AlbB on the fecundity of *An. stephensi*, we measured the number of eggs laid per LB1 female and the proportion of those eggs that hatched into larvae, as compared to wild-type LIS and the aposymbiotic line LBT mosquitoes. When mosquitoes were fed on mouse blood, there was no significant difference in the number of eggs laid by each female between the LB1 and LIS mosquitoes or between the LIS and LBT mosquitoes, although the LBT females laid slightly fewer eggs than did the LB1 females (P < 0.05, Student’s *t*-test) (Figure 1A). The egg hatch rate of the LB1 mosquitoes (50.0%) was significantly lower than that of either the LIS (73.0%; P < 0.0001, χ2 = 998.1) or LBT (74.6%; P < 0.0001, χ2 = 686.3) mosquitoes (Figure 1B). Similar results were observed when the mosquitoes were fed on sheep blood: LB1 and LIS mosquitoes laid a similar number of eggs, but the egg hatch rate of the LB1 mosquitoes (50.2%) was lower than that of the LIS mosquitoes (65.7%; P < 0.0001, χ2 = 42.7) (Figure 1C, D). We also compared the egg hatch rate of the LB1 and LBT mosquitoes after they had fed on human blood. Consistently, the number of eggs laid by the LB1 females was higher than that of the LBT mosquitoes (P < 0.0001) (Figure 1E), but the hatching rates of the LB1 mosquitoes (46.19%) were still significantly lower than those of the LBT strain (61.97%; P < 0.0001, χ2 = 119.7) (Figure 1F).

### *w*AlbB has no impact on the life history traits and sex ratio of the LB1 mosquitoes

In order to determine whether *w*AlbB influenced mosquito larvae survivorship and development, we measured 1) the survivorship from L1 larvae to pupae and adults, 2) the development time from L1 larvae to pupae and from pupae to adults, and 3) the adult sex ratio. As shown in Table 1, there was no significant difference between LB1 and LIS mosquitoes in survivorship during development from the L1 larval stage to pupae/adults. Both LB1 and LIS took a similar time to develop from L1 larvae to pupae and from pupae to adults. Furthermore, no significant difference was observed in the sex ratios of LB1 and LIS mosquitoes.

**Table 1:** **Life history attributes of *w*** **AlbB *Wolbachia-*** **infected and uninfected *An. stephensi***

**Attributes**	**LIS**	**LB1**	**P-value**
Survivorship from L1 to pupa, %	89.00 ± 3.21	89.33 ± 4.04	1
Pupation time, hr	205.05 ± 3.03	194.75 ± 11.78	0.2164
Survivorship from L1 to adult, %	80.67 ± 5.86	82.67 ± 4.16	0.655
Female ratio, %	55.34 ± 3.25	50.56 ± 11.64	0.7229
Male emergence time, hr	238.04 ± 5.79	227.68 ± 8.9	0.1485
Female emergence time, hr	244.68 ± 0.76	238.07 ± 7.56	0.2075

An assumption of a 1:1 sex ratio was made when larvae were placed into rearing pans. Immature data are based on three replicates. For pupation time, pupa emerging within 5 consecutive days were used for analysis. During this time, pupal emergence in each rearing pan was >85%. Very few of the larvae, which were still at 3^rd^ or 4^th^ instar at this time, were considered slow-growing and excluded from analysis. Fisher’s exact test was used for comparison of survivorship and sex ratio, and Student’s *t*-test was used for comparisons of development time.

### *w*AlbB has no impact on the body size of LB1 mosquitoes

**Figure 2:**
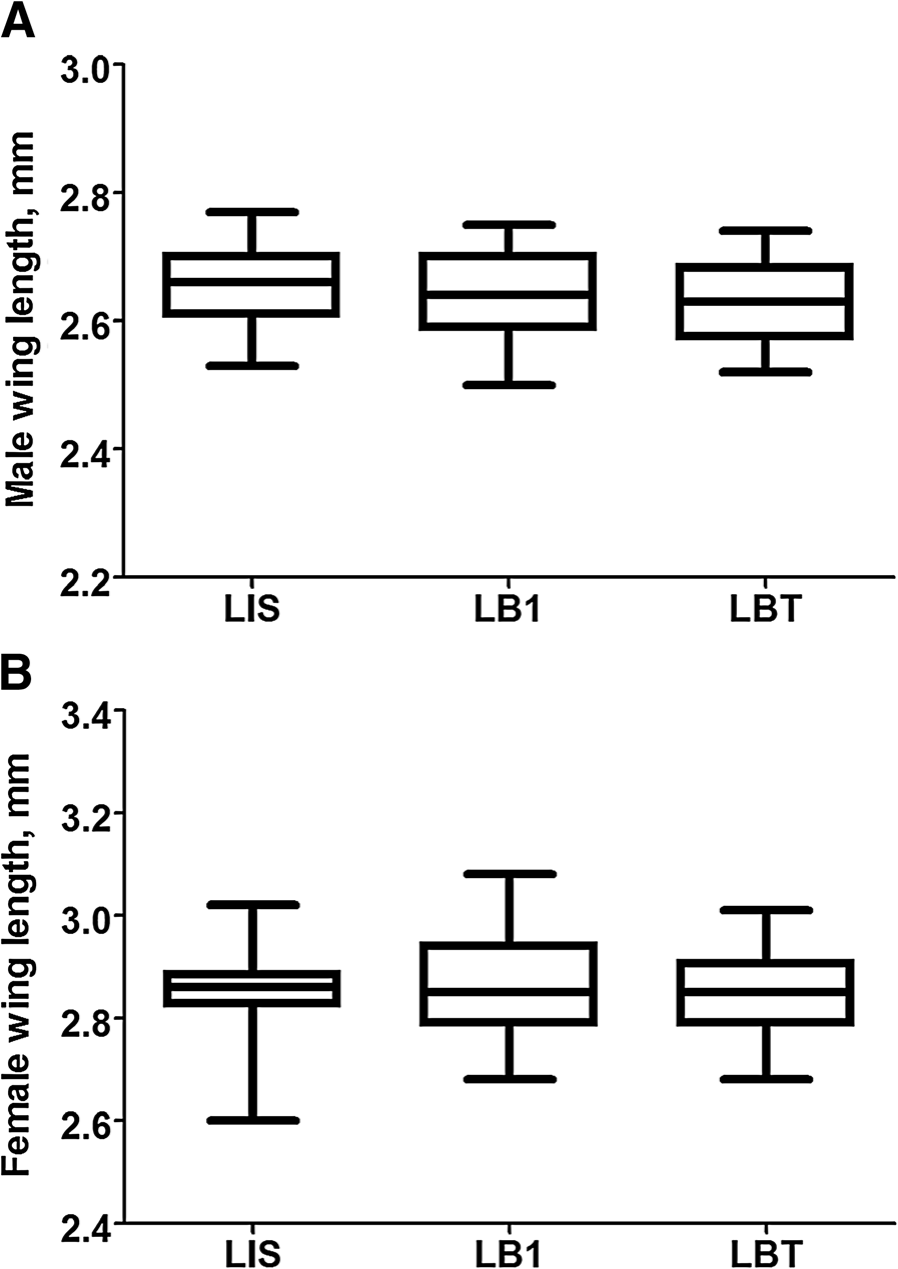
**Impact of *w*AlbB on the body size of *An. stephensi*.** Box plots display the observed distribution of wing length in female **(A)** and male **(B)** LIS, LB1, and LBT mosquitoes. The boxes in each panel represent (from bottom to top) the 25th to 75th percentiles. Horizontal bars within the boxes indicate the median value of each group. Interactions between *w*AlbB *Wolbachia* infection and body size, as based on measurement of the wing size, were not detected in either sex [P = 0.7746 for **(A)**; P = 0.487 for **(B)**; one-way ANOVA].

Insect body size reflects changes in the environment and can be an indicator of overall fitness. To determine whether *w*AlbB has any impact on the body size of LB1 mosquitoes, we measured the wing size of both females and males at 7-8 days old, after eclosion. We saw no significant difference in the wing size of the LIS, LB1, and LBT mosquitoes in both sexes (Figure 2A, B).

### *w*AlbB has a minor impact on LB1 males’ mating competition

**Figure 3:**
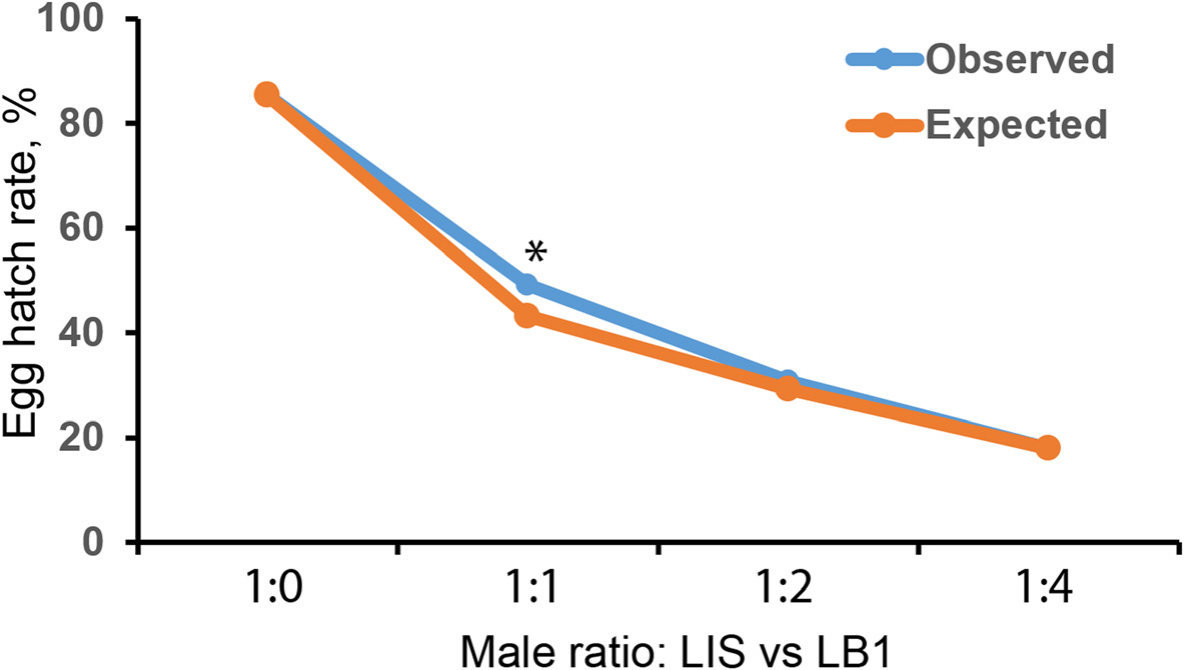
**Impact of *w*AlbB on male mating competitiveness.** Suppression of egg hatch in LIS populations via release of LB1 males. The blue line illustrates the egg hatch observed in population cage tests. The orange line illustrates the expected egg hatch, assuming equal competitiveness of LB1 and LIS males [24].

The ability of LB1 males to compete with LIS males for mating with LIS females is important for developing a population suppression strategy. Therefore, we investigated mating competition by using laboratory population cages. Four cages containing different ratios of LIS females to LIS males to LB1 males (1:1:0, 1:1:1, 1:1:2 and 1:1:4) were set up. When only LIS males were present, the egg hatch rate reached 85.3% (Figure 3; Additional file 1: Table S1). The expected egg hatch (Figure 3; Additional file 1: Table S1) was calculated assuming equal competitiveness of LB1 and LIS males [24] and based on an egg hatch rate of 1.2% in the CI matings [7]. A comparison between the observed egg hatch and the expected egg hatch (above) showed that there was no significant difference between the two groups at the ratios of 1:1:2 and 1:1:4. Only a minor (1.78%) deviation was observed at 1:1:1, but this difference is statistically significant (P < 0.001, χ2 = 11.7) (Figure 3; Additional file 1: Table S1).

### *w*AlbB increases longevity of sugar-fed LB1 mosquitoes but not blood-fed LB1 females

**Figure 4:**
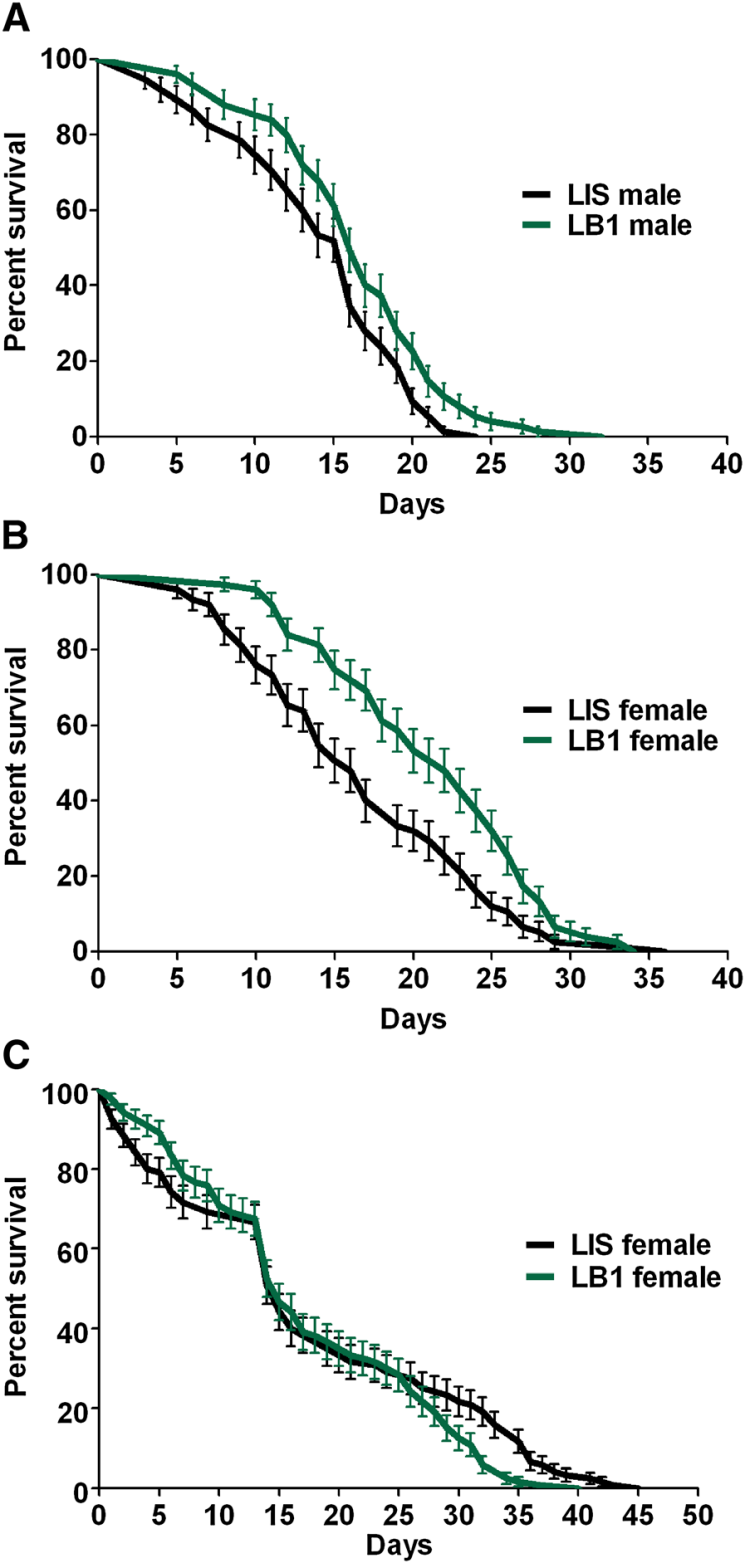
**Impact of *w*AlbB on the life span of *An. stephensi*.** Mosquitoes were provided with either 10% sucrose only **(A and B)** or blood meals to females **(C)**. One day after eclosion **(A and B)** or a blood meal **(C)**, males or females were individually transferred to mesh-covered cardboard buckets with 10% sucrose available. The dead mosquitoes were removed with an aspirator and recorded daily. The curves represent the mean percentage of mosquitoes surviving from three biological replicates each day. Both female and male LB1 mosquitoes lived significantly longer than did LIS mosquitoes (P = 0.0017 for **(A)**; P = 0.0095 for **(B)**; log-rank test) when maintained on sucrose alone. There was no significant difference in the life span of blood-fed females between LB1 and LIS mosquitoes **(C)**.

Previous studies have shown that *w*AlbB may provide a fitness advantage to mosquito hosts by increasing their longevity. To determine whether a similar impact would be produced in *An. stephensi*, we first compared the longevity of females (not blood-fed) and males between LB1 and LIS mosquitoes when they were maintained on 10% sucrose alone. We found that both sexes of LB1 mosquitoes lived significantly longer than did LIS mosquitoes (log-rank test, p < 0.01) (Figure 4A, B). Although both LB1 and LIS males had a median longevity of 16 days, the median LB1 female longevity (22 days) was 6 days longer than that of LIS females (16 days).We then compared the longevity of LB1 and LIS females after feeding on human blood. There was no significant difference between the two groups (log-rank test, P > 0.05). Both LB1 and LIS females had a median longevity of 15 days. LB1 females appeared to survive better during the first 10 days, whereas LIS females showed better survivorship later (after Day 26) (Figure 4C).

## Discussion

We have previously shown that the *Wolbachia* strain *w*AlbB forms a stable association with *An. stephensi*, invades laboratory populations of this mosquito, and induces resistance to *P. falciparum* in this mosquito vector [7]. To facilitate the development of *Wolbachia*-based strategies for malaria vector control, we examined *w*AlbB-associated fitness in *An. stephensi* in the present study. We found that *Wolbachia w*AlbB did not influence the egg numbers laid by LB1 females, but it reduced the egg hatch rate. Neither survivorship and development from L1 larvae to adults nor the adult sex ratio was influenced by *w*AlbB. *w*AlbB did have a very minor impact on the mating competitiveness of LB1 males. Longevity comparison experiments showed that both female and male *w*AlbB-infected *An. stephensi* lived significantly longer than their wild-type counterparts when the mosquitoes were fed on sugar meals alone. However, there was no difference in longevity between the LB1 and LIS females when they were given blood meals.

A reduction in egg hatch rate was consistently observed in LB1 mosquitoes fed mouse, sheep, or human blood. This result differed from that observed for *Ae. albopictus*, the original host of *w*AlbB, in which *Wolbachia* confers a reproduction advantage [16]. No fitness cost was seen when *w*AlbB was stably introduced into *Ae. aegypti *either [5], suggesting that this fitness-related effect is host-specific and is not only determined by the *Wolbachia* strain. A reduction in egg hatch rate was also observed in *Ae. aegypti* carrying a stable *w*MelPop infection, but mainly when the mosquitoes fed on nonhuman blood [25]; when they fed on human blood, however, only a mild decrease was observed [25]. We examined the hatch rate of LB1 eggs produced by females fed on human blood through an artificial feeding approach and found that the reduced egg hatch could not be restored, indicating that a different mechanism is responsible for the low egg hatch.

The reduction in LB1 egg hatch rate may be the result of an alteration in mosquito physiology, including changes in amino acids and ROS levels as a result of *Wolbachia* infection. A recent study has shown that the egg viability defect in *Wolbachia*-infected *Ae. aegypti* can be partially rescued by dietary supplementation with amino acids, supporting the possibility that the low egg hatch may be caused by a manipulation of amino acids [26]. For example, *Wolbachia* may compete with the mosquito host for amino acids, resulting in a lack of sufficient nutrition for embryonic mosquito development. Previous studies have also shown that a strain of *An. gambiae* that was genetically selected to be refractory to malaria parasites has significantly higher levels (2 to 3 times) of hemolymph H_2_O_2_ than do unselected strains [27]. However, this enhanced immunity to *Plasmodium* infection came with an adverse effect on fecundity, which could be restored by supplementation of mosquitoes with antioxidants [27]. Similarly, the ROS level in the fat body, midgut, and whole body of LB1 mosquitoes are significantly higher than LIS mosquitoes [7], and this increase is likely to be one of mechanisms that mediates refractoriness to malaria parasites. Because ROS detoxification by catalase is a major determinant of fecundity in the mosquito *An. gambiae*, the elevated ROS in LB1 mosquitoes may not be able to be cleared by catalase, resulting in a reduction in egg hatch. Future studies should determine whether supplementation of LB1 mosquitoes with amino acids and antioxidants can restore this decline in fecundity.

The impact of *Wolbachia* on the immature stage has been reported in various hosts [28,29], and a recent model has predicted that even those *Wolbachia* infections that cause minor decreases in immature survival are unlikely to invade and spread within the host population [30]. In *Aedes* mosquitoes, a negative impact of *Wolbachia* infections on larval survival and development time has been observed [28,29]. Here, we did not see any effect of *w*AlbB on survivorship during the development from L1 larvae to pupae/adults in LB1 versus LIS mosquitoes, consistent with the ability of *w*AlbB to invade laboratory *An. stephensi* population cages. However, our experiments were conducted at a relative low larval density. Previous studies have shown that *Wolbachia*-infected mosquito larvae experience reduced survival when intraspecific competition is intense [31,32]. It would be of interest to know whether a similar result would be obtained with LB1 mosquitoes.

Body size is an important indicator of a number of mosquito fitness traits [33,34]. With higher energy reserves, larger females may have a better flight range, higher survival, increased host-finding and blood-feeding success, and improved ability to locate oviposition sites, while larger males may live longer and have more mating success [35]. In addition, mosquitoes with a larger body size may take a larger blood meal, resulting in an increase in their intake of *Plasmodium* gametocytes and development of high infection intensity [36]. However, we saw no significant difference in the wing size in the LIS, LB1, and LBT mosquitoes. This result is similar to that of a previous report in *Ae. aegypti* that the differences in the body size can largely be attributed to nutrition and, to a minor extent, to *w*Mel *Wolbachia* infection [35].

Previous studies have indicated that mating competitiveness may be negatively affected in the transinfected male mosquitoes. After transfer of *w*Ri *Wolbachia* into *Ae. albopictus*, a reduction in mating competitiveness was observed in comparison to wild-type mosquitoes [37]. However, no significant difference was found between the competitiveness of the wildtype and transinfected males after *w*Mel was transferred to *Ae. albopictus*[17]. Thus, the impact on competitiveness may be related to the *Wolbachia* strain used. We observed a very minor negative effect of *w*AlbB on mating competitiveness between LB1 and wild-type males only at the ratio of 1:1:1. When more LB1 males were released, with the ratio increased to 1:1:2 and 1:1:4, the observed egg hatch rate was not significantly different from the expected values, assuming an equal competitiveness of LB1 and LIS males, and suggesting that there is no significant difference in mating capacity between LB1 and LIS males. Together with the ability of LB1 males to induce nearly complete CI when mating with wild-type females, this strong mating competitiveness supports the prediction that mass release of LB1 males could lead to a potential local eradication of *An. stephensi*. This population suppression strategy has been successful in controlling *Culex* and *Aedes* mosquitoes in the field [21,22].

We found that *w*AlbB *Wolbachia* increased the longevity of *An. stephensi* in both sexes when mosquitoes were maintained on sucrose alone; however, there was no difference in the longevity of infected and uninfected females after they took a blood meal. As the original native host of *w*AlbB, *Ae. albopictus* lives significantly longer than the aposymbiotic line [16]. When *w*AlbB is transferred into and forms a stable association with *Ae. aegypti*, an increase in the longevity of the transinfected line is also observed [10]. An impact of *Wolbachia* on host longevity has also been found in other strains. Transfer of *w*Mel into *Ae. albopictus* leads to an increase in longevity only in the male, whereas *w*MelPop decreases the longevity of both native *Drosophila* and transinfected *Aedes*[17-19]. Thus, *Wolbachia*-host interactions must influence a mechanism that can determine host longevity. For example, the insulin/IGF-I signaling pathway is an evolutionarily conserved mechanism of longevity that is present from yeast to humans [38]. In *Drosophila*, *Wolbachia* has been reported to increase the fly’s insulin/IGF-like signaling [39]. It would be interesting to know whether *Wolbachia* changes the host’s life span by modulating the insulin/IGF-I signaling pathway.

The longevity of the mosquito vector contributes significantly to its vector capacity for malaria transmission. Because malaria parasites need to complete their development through an extrinsic incubation period in mosquitoes before being transmitted to humans, the older mosquitoes are more dangerous from the human perspective because they play more important roles in disease transmission. Although we observed an increase in the life span of LB1 females when they were maintained on sucrose alone, the LB1 females did not live significantly longer than the LIS females after taking human blood. In particular, LB1 females showed a trend toward living a shorter time than LIS females after Day 26. Additional experiments should be conducted to determine whether there is a change in the longevity of LB1 females after feeding on parasite-infected blood. It also should be noted that *Wolbachia* may clear all the parasites through its pathogen interference mechanism and leave all the older mosquitoes free of parasites. This possibility is supported by our recent observation that the *Wolbachia* density increases when LB1 mosquitoes age (data not shown).

On the other hand, the fitness advantage seen in the life span of LB1 mosquitoes that had not been blood-fed could have a positive impact on control strategies. For example, the LB1 females may survive better than LIS females do in an environment in which blood resources are limited or temporarily unavailable. This effect may counteract the negative impact of their low egg hatch, reduce the initial release threshold, and accelerate population replacement, resulting in improved efficacy when it is deployed as a practical malaria intervention strategy. Furthermore, only males will be released in the planned population suppression strategy. The increased longevity in the transinfected males may improve their mating capacity, resulting in more CI matings and stronger suppressive effects.

## Conclusions

The ability to stably introduce *w*AlbB into *An. stephensi* makes it possible to develop *Wolbachia*-based strategies for malaria vector control. Similar strategies have been successfully tested in field settings, and progress is being made toward eliminating dengue and lymphatic filariasis in a number of disease-endemic countries [6,22]. Both this prior experience and mathematical models suggest that the host fitness associated with *Wolbachia* is one of the key factors that determines the population dynamics following its field release. Our results have shown that *w*AlbB induces both a fitness cost and benefits in *An. stephensi* under laboratory conditions. All the data we have now accumulated for LB1 males, including their strong mating competitiveness, increased life span when taking sugar meals, and ability to induce nearly complete CI, support the feasibility of a population suppression/eradication strategy. While LB1-mediated population replacement has been demonstrated in the laboratory condition [7], the LB1 fitness data reported here could facilitate the development of an improved mathematic modeling guiding the next field trial in the malaria endemic areas where *An. stephensi* is a primary vector. However, the laboratory measures used here, while informative, may not exactly reflect the realities of a competitive field environment. There may be difference in the fitness associated with *Wolbachia* infection between the long-established laboratory colonies and the field mosquitoes. Future studies should focus on whether *Wolbachia*-associated fitness is subject to changes in environmental or field conditions, such as temperature, humidity, and blood source; how these changes can influence the outcome of malaria control; and how all this information can be utilized to develop better control strategies.

## Competing interests

The authors declare that they have no competing interests.

## Authors’ contributions

DJ and ZX designed research; DJ, MJM, DB and FZ conducted experiments; DJ and ZX. analyzed data and wrote the paper. All authors read and approved the final version of the manuscript

## Supplementary Material

Additional file 1: Table S1 Mating competitiveness between *w*AlbB-infected and uninfected *An. stephensi* males. Click here for file
