# Supplementary material for: Wolbachia strain wAlbB confers both fitness costs and benefit on Anopheles stephensi
Source: Parasit Vectors. 2014 Jul 21;7:336. doi: 10.1186/1756-3305-7-336 (PMC4223616; doi:10.1186/1756-3305-7-336)
Supplement: Additional file 1: Table S1 — Mating competitiveness between wAlbB-infected and uninfected An. stephensi males. [file 1756-3305-7-336-S1.docx]

**Additional file**

**Additional_file_1 as DOCX**
**Additional file 1: Table S1.** Mating competitiveness between *w*AlbB-infected and uninfected *An. stephensi* males

| Adult ratio |  | Adult number |  |  |  |  | | |
| --- | --- | --- | --- | --- | --- | --- | --- | --- |
| LIS (F): LIS (M): LB1(M) |  | LIS (F), LIS (M), LB1 (M) | Eggs scored | Observed hatching | Expected hatching* | | P value# |  |
| 1:1:0 |  | 100 (50,50,0) | 3243 | 85.32 | 84.90 | | ND |  |
| 1:1:1 |  | 105 (35,35,35) | 1689 | 50.08 | 43.23 | | 0.0006 |  |
| 1:1:2 |  | 200 (50,50,100) | 2940 | 28.70 | 29.20 | | 0.21 |  |
| 1:1:4 |  | 300 (50,50,200) | 2990 | 17.36 | 17.99 | | 0.92 |  |

* The expected egg hatch was calculated assuming equal competitiveness of LB1 and LIS males [[23](#_ENREF_24)].

# Comparisons between the observed and expected egg hatch through chi-squared test.
